# Analysis of the current status and associated factors of tuberculosis knowledge, attitudes, and practices among elderly people in Shenzhen: a cross-sectional study

**DOI:** 10.1186/s12889-021-11240-7

**Published:** 2021-06-17

**Authors:** Yunxia Wang, Yong Gan, Juanjuan Zhang, Jinzhou Mei, Jing Feng, Zuxun Lu, Xin Shen, Meigui Zhao, Yanfang Guo, Qing Yuan

**Affiliations:** 1 Shenzhen Bao’an Center for Chronic Disease Control, NO. 332 Yu’an 2nd Road, Shenzhen, 518101 China; 2 Department of Social Medicine and Health Management, School of Public Health, Tongji Medical College, Huazhong University of Science and Technology, Wuhan, Hubei China

**Keywords:** Tuberculosis, Knowledge, Attitude, Practice, Elderly people

## Abstract

**Background:**

The incidence and risk of tuberculosis (TB) among the elderly population have increased with the ageing population in China. This study aimed to assess the current status and associated factors of TB knowledge, attitudes, and practices among elderly people in Shenzhen City, China, which may provide references for the development of TB prevention and treatment policies targeting elderly people.

**Methods:**

A multistage random sampling method was used to collect data with a self-designed questionnaire from 1078 elderly people (response rate, 90.66%) living in Bao’an District of Shenzhen between September and October 2019. Univariate and multiple linear regression analyses were used to analyse factors associated with TB knowledge, attitudes, and practices among elderly people.

**Results:**

Among the respondents, 3.13% had previously been treated for TB, and 3.09% of respondents had family members or friends with a previous TB history. The percentages of elderly people who were aware of TB and had positive attitudes and practices regarding TB were 69.23%, 48.87%, and 42.62%, respectively. Multiple linear stepwise regression analysis showed that elderly people with a junior high school education or higher, a family annual income per capita of less than 100,000 RMB, a better self-perceived health status, and family members or friends with a previous TB history had higher TB knowledge scores (*P* < 0.05). Elderly people with medical insurance, a junior high school or higher education, a family annual income per capita of less than 100,000 RMB, and family members and friends with a previous TB history had higher TB attitude scores (*P* < 0.05). In addition, elderly people who were older, had medical insurance, had a junior high school education or higher, and had a family annual income per capita less than 100,000 RMB had higher TB practice scores (*P* < 0.05).

**Conclusions:**

Elderly people were aware of TB, but their positive attitudes and practices were at a low level. Corresponding prevention and treatment policies should be developed according to these influencing factors to reduce the incidence of TB among elderly people and improve their quality of life.

## Background

The Global Tuberculosis (TB) Report issued by the World Health Organization (WHO) showed approximately 10 million new TB patients worldwide in 2019, 86% of whom were concentrated in 30 countries with a high TB burden [1]. China has a high TB burden, with approximately 833,000 new TB patients every year, ranking second in the world behind India [1]. The TB epidemic is growing markedly, with a progressive increase in the notification rate with age, and a peak among those aged 65 years or older [1]. As the population has aged rapidly in China in recent years, the WHO estimates that the number of elderly people in China will reach 402 million by 2040, accounting for 28% of the total population [2]. A cohort study in 2020 showed that the incidence and risk of TB among the elderly population increased with the ageing of the population in China, and the detection rate of TB among the elderly population was 481.8/100,000 [3]. The high incidence of TB among elderly people has introduced considerable challenges to the lives of Chinese people and China’s economy. Understanding the knowledge, attitudes, and practices (KAP) and associated factors related to TB among elderly people is very important to the targeted development of TB prevention and treatment tasks in China.

Previous studies have investigated TB KAP among medical interns, nurses, teacher trainees, healthcare workers, and community residents [4–8]. Berg-Johnsen et al. conducted a study on 270 medical interns and found that the surveyed interns had an adequate level of TB-related knowledge and acceptable attitudes [4]. Akande reported that small proportions of the respondents had a good score for knowledge (10.5%) and practices (6%) [5]. Dorji et al. surveyed 420 trainees and revealed that 58.6% had low knowledge of TB [6]. Alotaibi et al. conducted a study of 540 healthcare workers and found that the participants had average knowledge, above-average attitude, and good practice regarding TB [7]. Wang et al. conducted a national survey of 33,357 residents and showed an overall TB awareness rate of 74.45% [8]. However, no studies have investigated TB KAP and associated factors among elderly people in the community. Therefore, this study aimed to address this important research gap. The findings from this study may contribute to developing TB prevention and treatment policies for elderly people.

## Methods

### Study population and sampling

Multistage random sampling was performed between September and October 2019. First, two of eight community health service centres with chest X-ray film screening capabilities in Bao’an District of Shenzhen were randomly selected. Next, six hundred people aged 65 years or older who received health examinations were randomly selected from each community health service centre. Elderly people in the study communities who met the following criteria were included in the survey: (1) aged 65 years and older; (2) residence in the area for at least half a year; (3) no communication disorders or mental illnesses; and (4) willingness to complete the survey. Individuals were excluded if they did not meet one of the above requirements.

The sample size was calculated using the formula, n = [Z^2^p(1 − p)]/d^2^ (where n = sample size, Z = confidence level for a normal distribution, p = estimated proportion, and d = absolute error) [9]. Taking a confidence interval (*CI*) of 95% with a probability of 51.4% [10] and a margin of error at 5%, the sample size was rounded off to 400. To compensate for nonresponses, the sample size was increased by 10% to 440. Thus, the sample size was sufficient in our study.

This study was approved by the ethics committee of Tongji Medical College institutional review board, Huazhong University of Science and Technology, Wuhan, China. All methods were carried out in accordance with relevant guidelines and regulations.

### Questionnaire design and measurement

A cross-sectional study was performed to collect data from the elderly population using a structured questionnaire. The survey questionnaire was designed according to core information on TB prevention and treatment in China (2016 edition) [11], available questionnaires in previous literatures [10, 12, 13], and the actual conditions of elderly people in Shenzhen city. The questionnaire included two parts; the first part collected information on gender, age, residence, education level, marital status, occupation, medical insurance, family annual income per capita, self-perceived health status, body mass index (BMI), tobacco use, alcohol consumption, previous TB history, and family members or friends with a previous TB history. The second part examined the subjects’ KAP regarding the prevention and treatment of TB. TB KAP was examined with 5, 5, and 6 items respectively, including TB epidemiology, transmission, treatment, and infection prevention and control. Incorrect/inappropriate or uncertain (did not know) responses were given a score of 0, while 1 point was assigned for selecting the correct/appropriate answer; correct/appropriate responses were based on current literature and best practice. Respondents who answered 60% of the KAP questions correctly were considered aware of TB and to have positive attitudes or practices regarding TB.

### Data collection and quality control

We designed the questionnaire based on a literature review, group discussions, and expert consultation. To improve the quality of the questionnaire, a pretest was conducted at Shenzhen’s community health service centres. Then, trained investigators administered the questionnaire to elderly people. Questionnaires were recovered on-site and the completeness of the questionnaires was verified. The data were entered into the database in a double-blind manner by two different researchers using EpiData 3.1 to guarantee accuracy.

### Statistical analysis

All statistical analyses were performed using Statistical Package for Social Sciences (SPSS, Inc., Chicago, IL, Version 13.0). Descriptive analyses used the mean, standard deviation (SD), median, and interquartile range (IQR) for continuous variables and percentages for categorical data. Univariate linear regression analysis was performed to identify associations between different predictive variables and TB KAP among elderly people. Multiple linear stepwise regression was used to estimate factors associated with TB KAP among elderly people (levels for selection and elimination: *P* = 0.05 and *P* = 0.10, respectively). TB KAP scores were used as dependent variables, and the predictive variables included age, BMI, gender, residence, education level, marital status, occupation, medical insurance, annual family income per capita, self-perceived health status, tobacco use, alcohol consumption, previous TB history, and family members or friends with a previous TB history.

## Results

**Fig. 1:**
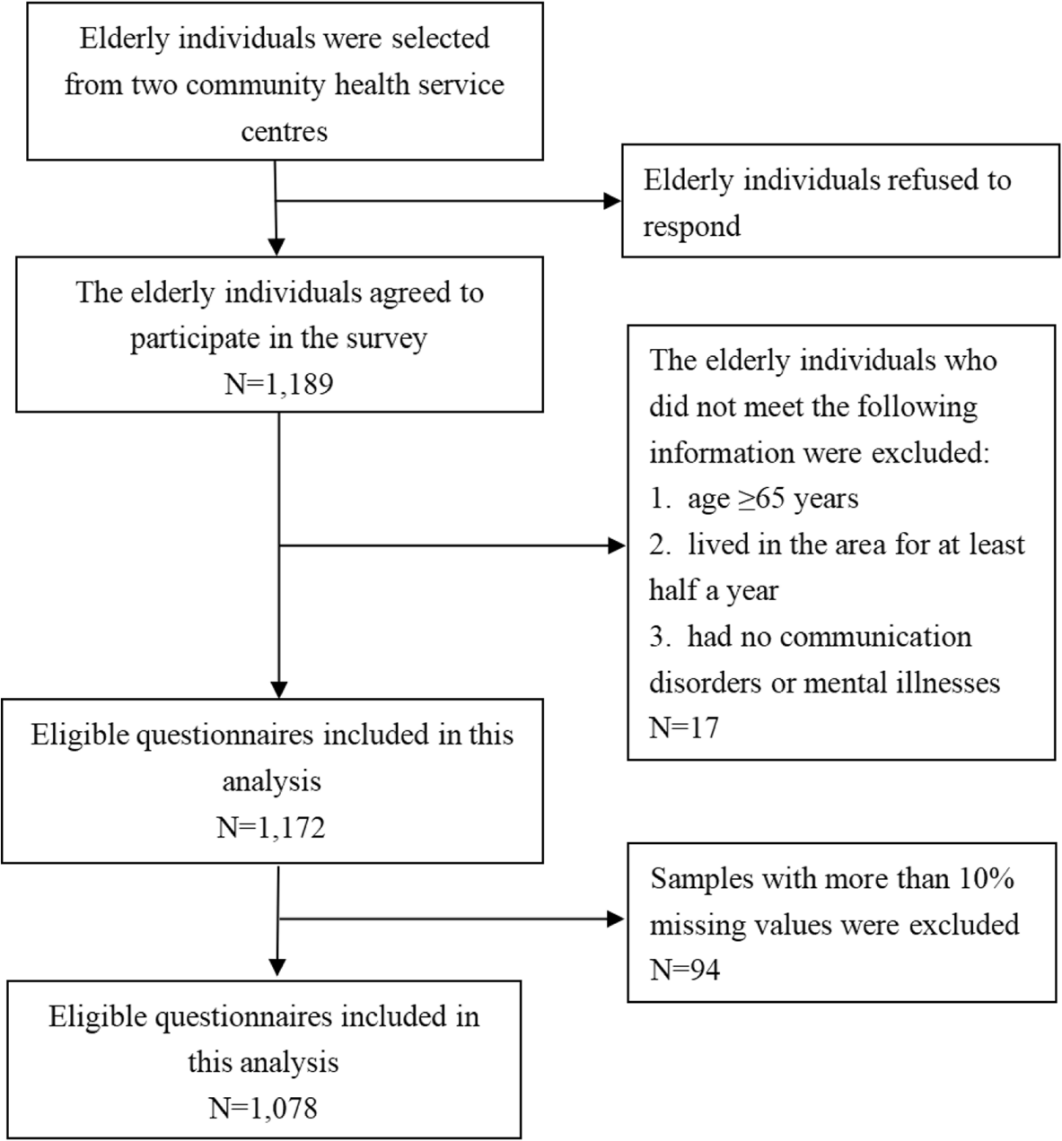
The flow chart for sampling in this study

Initially, a total of 1200 elderly people were randomly recruited for the survey. Eleven elderly people refused to participate and 1189 completed questionnaires were received. Then, we excluded 17 interviewees aged < 65 years, residing in the area for less than half a year, and with communication disorders and mental illnesses. During the statistical analysis, 94 samples with more than 10% missing values for the items of knowledge, attitudes, and practices were excluded. Finally, a total of 1078 samples were included in this study, yielding a response rate of 90.66% (see Fig. 1).

Table 1 shows the basic characteristics of elderly people. Among 1078 respondents, 488 (45.40%) were females, and 587 (54.60%) were males. Most elderly people (77.68%) were nonlocal residents. A total of 582 (54.65%) respondents had a primary school education or below. Most (89.37%) elderly people were married, 80.95% were farmers or worked in other occupations and 72.89% had medical insurance. Elderly people with a family annual income per capita lower than 100,000 RMB accounted for 71.44% of the total sample. A total of 494 (47.45%) and 481 (46.21%) respondents reported a good or fair self-perceived health status respectively. A total of 118 (10.97%) and 100 (9.28%) elderly individuals were current cigarette smokers or alcohol drinkers, respectively. Among the population, 33 (3.13%) had previously been treated for TB, and 33 (3.09%) had family members or friends with a previous TB history. In addition, the average age of the elderly individuals was 71.42 (SD = 5.22) years, and the average BMI was 23.67 (SD = 3.33) kg/m^2^.

**Table 1:** Characteristics of the study populations

Variables		n/mean	%/SD
Total		1078	100.00
Age (years)		71.42	5.22
BMI (kg/m^2^)		23.67	3.33
Gender	Male	488	45.40
	Female	587	54.60
Residence	Local residents	237	21.99
	Others	825	77.68
Education level	Primary school or lower	582	54.65
	Junior high school or higher	483	45.35
Marital status	Married	933	89.37
	Unmarried/widowed/divorced	111	10.68
Occupation	Workers or administrators	204	19.05
	Farmers or others	867	80.95
Medical insurance	Yes	769	72.89
	No	286	27.11
Family annual income per capita (RMB)	Less than 100,000	743	71.44
	More than 100,000	297	28.56
Self-perceived health status	Good	494	47.45
	Fair	481	46.21
	Bad	66	6.34
Cigarette smoker	Current smoker	118	10.97
	Ex/non-smoker	958	89.03
Alcohol drinker	Current drinker	100	9.28
	Ex/non-drinker	978	90.72
Previously treated for TB	Yes	33	3.13
	No	1020	96.87
Family members or friends with a previous TB history	Yes	33	3.09
	No	1036	96.91

Abbreviations: SD = standard deviation, BMI = body mass index, TB = tuberculosis

The mean TB KAP scores were 3 (IQR = 2; range 0 to 5), 2 (IQR = 1; range 0 to 5), and 3 (IQR = 2; range 0 to 6). The percentages of elderly people who were aware of TB and had positive attitudes and practices were 69.23%, 48.87%, and 42.62% respectively.

The distribution of participants’ responses regarding TB KAP is shown in Table 2. In the knowledge section, more than 70% of the elderly individuals (72.60%, 73.94%, and 71.62%) were aware of TB infectivity, symptoms, and preventive measures, respectively. The awareness rate for TB curability was 45.30%. However, less than one-third of survey respondents (31.03%) knew the route of TB transmission. In terms of attitudes, most elderly people (86.03% and 86.89%) wanted to learn about TB or participate in TB education activities, respectively. More than one-third of respondents (36.44%) were willing to be screened for TB if they had suggestive symptoms. Only 225 (21.15%) and 261 (24.90%) participants did not think that TB is a terrible disease or were willing to complete treatment if diagnosed with TB. Regarding practices, most elderly individuals (84.08% and 85.69%) would visit health facilities if they had a cough for more than 2 weeks or if they suspected that they had TB, respectively. More than half of elderly people (60.54%) would urge their friends with suspicious symptoms to visit the doctor. Approximately one-third of participants (33.08% and 33.02%) had ever taken the initiative to learn about TB or would stop spitting in public, respectively. Only 265 (25.09%) respondents covered their mouths and noses when coughing or sneezing.

**Table 2:** The distribution of participants’ responses regarding TB KAP (*n* = 1078)

Items	Responses	n	%
**Knowledge**			
Infectivity of TB			
	Yes	779	72.60
	No	83	7.74
	Do not know	211	19.66
Route of TB transmission			
	Touching items	85	7.94
	Sharing utensils	117	10.93
	Coughing or sneezing	332	31.03
	All of the above	319	29.81
	Do not know	217	20.28
TB symptoms			
	Headache or dizziness	25	2.35
	Coughing for longer than 2 weeks or coughing up blood	786	73.94
	Abdominal pain or diarrhoea	5	0.47
	Do not know	247	23.24
Prevention and control of TB			
	Covering mouth and nose when coughing or sneezing	184	17.18
	Wearing a mask	51	4.76
	Good nutrition	21	1.96
	Washing hands after touching items in public	48	4.48
	All of the above	767	71.62
Curability of TB			
	Yes	477	45.30
	No	277	26.31
	Do not know	299	28.40
**Attitudes**			
Do you think that TB is a terrible disease?			
	Yes	646	60.71
	No	225	21.15
	Do not sure	193	18.14
Would you like to learn about TB?			
	Yes	924	86.03
	No	15	1.40
	Do not sure	135	12.57
Would you like to participate in TB education activities?			
	Yes	921	86.89
	No	26	2.45
	Do not sure	113	10.66
Are you willing to complete treatment if you have TB?			
	Yes	261	24.90
	No	410	39.12
	Do not sure	377	35.97
Would you be willing to be screened for TB if you had suggestive symptoms?			
	Yes	387	36.44
	No	494	46.52
	Do not sure	181	17.04
**Practices**			
Have you ever taken the initiative to learn about TB?			
	Yes	354	33.08
	No	716	66.92
Would you urge your friends with suspicious TB symptoms visit the doctor?			
	Yes	649	60.54
	No	335	31.25
	Do not sure	88	8.21
Would you visit a health facility if you had a cough for more than 2 weeks?			
	Yes	903	84.08
	No	131	12.20
	Do not sure	40	3.72
Would you visit a health facility if you suspected that you had TB?			
	Yes	916	85.69
	No	132	12.35
	Do not sure	21	1.96
Would you stop spitting in public?			
	Yes	313	33.02
	No	580	61.18
	Do not sure	55	5.80
Would you cover your mouth and nose when coughing or sneezing?			
	Yes	265	25.09
	No	791	74.91

Abbreviations: TB = tuberculosis, KAP = knowledge, attitudes, and practices

Table 3 presents the results of the univariate linear regression analysis of TB KAP among the elderly population. Significant differences in TB knowledge among elderly people were observed across education levels, medical insurance statuses, family annual incomes per capita, self-perceived health statuses, previous TB histories, and family members or friends with a previous TB history (*P* < 0.05). Significant differences in TB attitudes among elderly people were found across BMIs, education levels, occupations, medical insurance statuses, family annual incomes per capita, previous TB histories, and family members or friends with a previous TB history (*P* < 0.05). Significant differences in TB practices among elderly people were identified across ages, residences, education levels, occupations, medical insurance statuses, family annual incomes per capita, self-perceived health statuses, previous TB histories, and family members or friends with a previous TB history (*P* < 0.05).

**Table 3:** Univariate linear regression analysis of KAP regarding TB among the elderly population

Variables	Knowledge			Attitudes			Practices		
	***B***	95% ***CI***	***P***	***B***	95% ***CI***	***P***	***B***	95% ***CI***	***P***
Age (years)	0.002	−0.012 to 0.017	0.771	0.007	−0.006 to 0.021	0.305	0.022	0.004 to 0.040	0.020
BMI (kg/m^2^)	−0.017	−0.040 to 0.005	0.137	−0.022	−0.043 to −0.001	0.036	−0.019	−0.047 to 0.008	0.167
Gender (ref. male)	−0.090	−0.243 to 0.063	0.247	−0.054	−0.194 to 0.087	0.453	0.036	−0.147 to 0.220	0.698
Residence (ref. local residents)	−0.108	−0.293 to 0.076	0.248	−0.003	−0.174 to 0.169	0.975	−0.334	−0.558 to −0.110	0.003
Education level (ref. primary school or lower)	0.286	0.133 to 0.438	<0.001	0.275	0.135 to 0.415	<0.001	0.318	0.134 to 0.503	0.001
Marital status (ref. married)	−0.208	−0.279 to 0.222	0.825	0.168	−0.060 to 0.396	0.149	0.217	−0.076 to 0.509	0.147
Occupation (ref. workers or administrators)	−0.119	−0.313 to 0.075	0.230	−0.331	−0.488 to −0.134	0.001	−0.381	−0.617 to −0.144	0.002
Medical insurance (ref. yes)	−0.260	−0.432 to −0.088	0.003	−0.467	−0.624 to −0.310	<0.001	−0.563	−0.763 to −0.363	<0.001
Family annual income per capita (RMB) (ref. less than 100,000)	−0.229	−0.470 to −0.128	0.001	−0.300	−0.457 to −0.143	<0.001	−0.458	−0.664 to −0.251	<0.001
Self-perceived health status	−0.211	−0.338 to −0.084	0.001	−0.063	−0.180 to 0.055	0.298	−0.192	−.342 to −0.041	0.013
Cigarette smoker (ref. current smoker)	−0.113	−0.357 to 0.130	0.360	−0.152	−0.377 to 0.073	0.186	−0.031	−0.327 to 0.265	0.837
Alcohol drinker (ref. current drinker)	0.054	−0.208 to 0.315	0.686	0.024	−0.217 to 0.264	0.846	0.007	−0.320 to 0.334	0.966
Previously treated for TB (ref. no)	0.668	0.235 to 1.101	0.003	0.406	0.002 to 0.810	0.049	0.755	0.232 to 1.278	0.005
Family members or friends with a previous TB history (ref. no)	0.709	0.272 to 1.147	0.002	0.829	0.432 to 1.225	<0.001	0.448	−0.065 to 0.962	0.087

Abbreviations: KAP = knowledge, attitudes, and practices, TB = tuberculosis, BMI = body mass index

* The self-perceived health status variable was rated as follows: good = 1, fair = 2, bad = 3

Multiple stepwise regression analyses showed that education level (junior high school or higher: *β* = 0.278, 95% *CI* 0.108 to 0.447; *P* = 0.001), annual family income per capita (more than 100,000: *β* = − 0.377, 95% *CI* − 0.563 to − 0.191; *P* < 0.001), self-perceived health status (*β* = − 0,245, 95% *CI* − 0.387 to − 0.103; *P* = 0.001), and having family members or friends with a previous TB history (yes: *β* = 0.624, 95% *CI* 0.157 to 1.091; *P* = 0.009) were associated with TB knowledge among elderly people (Table 4). Medical insurance (no: *β* = − 0.409, 95% *CI* − 0.584 to − 0.235; *P* < 0.001), education level (junior high school or higher: *β* = 0.208, 95% *CI* 0.052 to 0.364; *P* = 0.009), family annual income per capita (more than 100,000: *β* = − 0.224, 95% *CI* − 0.394 to − 0.053; *P* = 0.010), and having family members or friends with a previous TB history (yes: *β* = 0.753, 95% *CI* 0.332 to 1.174; *P* < 0.001) were associated with TB attitude scores (Table 5). In addition, age (*β* = 0.028, 95% *CI* 0.007 to 0.048; *P* = 0.007), medical insurance (no: *β* = − 0.494, 95% *CI* − 0.716 to − 0.272; *P* < 0.001), education level (junior high school or higher: *β* = 0.331, 95% *CI* 0.127 to 0.536; *P* = 0.002), and family annual income per capita (more than 100,000: *β* = − 0.340, 95% *CI* − 0.564 to − 0.117; *P* = 0.003) were associated with TB practice scores (Table 6).

**Table 4:** Multiple linear stepwise regression analysis of TB knowledge TB among the elderly population

Variables	***B***	***SE***	***Beta***	***t***	***P***	95% ***CI***
Education level (ref. primary school or lower)						
Junior high school or higher	0.278	0.086	0.109	3.210	0.001	0.108 to 0.447
Family annual income per capita (RMB) (ref. less than 100,000)						
More than 100,000	−0.377	0.095	−0.135	−3.973	< 0.001	−0.563 to − 0.191
Self-perceived health status	−0.245	0.072	−0.115	−3.379	0.001	−0.387 to − 0.103
Family members or friends with a previous TB history (ref. no)						
Yes	0.624	0.238	0.088	2622	0.009	0.157 to 1.091
Intercept	3.413	0.225		15.147	< 0.001	2.971 to 3.855

Abbreviations: TB = tuberculosis, SE = standard error

* The self-perceived health status variable was rated as follows: good = 1, fair = 2, bad = 3

**Table 5:** Multiple linear stepwise regression analysis of TB attitudes among the elderly population

Variables	***B***	***SE***	***Beta***	***t***	***P***	95% ***CI***
Medical insurance (ref. yes)						
No	−0.409	0.089	−0.160	−4.619	< 0.001	−0.584 to − 0.235
Education level (ref. primary school or lower)						
Junior high school or higher	0.208	0.079	0.089	2.615	0.009	0.052 to 0.364
Family annual income per capita (RMB) (ref. less than 100,000)						
More than 100,000	−0.224	0.087	−0.088	−2.571	0.010	−0.394 to − 0.053
Family members or friends with a previous TB history (ref. no)						
Yes	0.753	0.214	0.118	3.514	< 0.001	0.332 to 1.174
Intercept	3.016	0.193		15.646	< 0.001	2.638 to 3.395

Abbreviations: TB = tuberculosis, SE = standard error

* The self-perceived health status variable was rated as follows: good = 1, fair = 2, bad = 3

**Table 6:** Multiple linear stepwise regression analysis of TB practice among the elderly population

Variables	***B***	***SE***	***Beta***	***t***	***P***	95% ***CI***
Age (years)	0.028	0.010	0.096	2.692	0.007	0.007 to 0.048
Medical insurance (ref. yes)						
No	−0.494	0.113	−1.161	−4.375	< 0.001	−0.716 to − 0.272
Education level (ref. primary school or lower)						
Junior high school or higher	0.331	0.104	0.115	3.181	0.002	0.127 to 0.536
Family annual income per capita (RMB) (ref. less than 100,000)						
More than 100,000	−0.340	0.114	−0.108	−2.993	0.003	−0.564 to − 0.117
Intercept	1.800	0.799		2.252	0.025	0.231 to 3.370

Abbreviations: TB = tuberculosis, SE = standard error

* The self-perceived health status variable was rated as follows: good = 1, fair = 2, bad = 3

## Discussion

Previous studies have shown that TB KAP were directly associated with the detection rate and TB treatment and prevention effects [14]. The results showed that the core knowledge awareness rate for TB prevention and treatment among elderly people in Shenzhen City was 69.23%, which is higher than the rates reported for residents of Zhejiang Province (48.0%) [15], Guizhou Province residents (41.5%) [16], and hospitalized elderly TB patients in Hunan (56.2%) [17]. However, this rate is considerably lower than the nationwide public TB knowledge awareness rate reported in 2009 (89.0%) [18]. In terms of each key piece of information about TB, the elderly people had the highest level of correct knowledge about TB’s symptoms, which was much better than participants in Lesotho [20]. But what is noteworthy is that a small proportion of respondents knew the transmission routes and curability of TB, which should be strengthened in health education. The percentages of elderly people with positive attitudes and practices regarding TB were 48.87 and 42.62%, respectively, which are slightly higher than the positive attitude rate (40.8%) and lower than the positive practice rate (45.9%) found in residents of Ethiopia [19] and considerably lower than the incidence of positive attitudes reported by Luba et al. [20] for residents of Lesotho (72.8%). Therefore, implementing further TB health promotion and education efforts among elderly people in the community is an urgent task for the government and related sectors to improve TB prevention and treatment for elderly people.

The results of the multiple linear stepwise analyses showed that the TB knowledge scores of elderly people with higher education levels, lower family annual incomes per capita, a better self-perceived health status, and family members or friends with a previous TB history were higher. Elderly people with a junior high school education or above had higher TB knowledge scores, which is consistent with the findings of Chen et al. [15], Wan [21], and Wang et al. [22]. Elderly individuals with higher annual family incomes per capita had lower TB knowledge scores, which is inconsistent with previous studies [23]. One possible reason is that older individuals have different concerns about health knowledge, and high-income groups may be more knowledgeable in other areas of health knowledge [24]. In addition, because the percentage of people with higher self-reported annual family incomes was relatively low in this study and residents may be sensitive about sharing income information, the number of people with high incomes may have been underestimated. Intriguingly, this study showed that elderly individuals with higher education levels were more likely to have higher levels of TB knowledge, but individuals with higher incomes were less likely to have TB knowledge, reflecting an interesting finding that requires further research. Educational interventions should be strengthened for elderly people with low education levels and high incomes. Furthermore, this study showed that people with a better self-perceived health status had higher TB awareness. Elderly people with a better self-perceived health status may direct more attention towards health information and their own health; therefore, they were more likely to acquire more TB information. Elderly people whose relatives or friends previously had TB also had more opportunities to be informed about TB and were strongly influenced by its importance. Therefore, they reported higher awareness rates, which also suggests that some individuals successfully received information from the health care system and spread health knowledge. Therefore, health education activities are needed in medical institutions to further health knowledge among the elderly population.

The TB attitude scores of elderly individuals without health insurance were lower. Elderly people with higher education levels and those whose family members or friends with a previous TB history had relatively positive attitudes towards TB treatment and prevention. The impact of education level on TB attitude and practices scores was consistent with the impact of knowledge scores, indicating that health education regarding TB was the key to establishing appropriate health attitudes and practices. Our finding is consistent with the study by Luba et al. [20] among Lesotho residents. In addition, age was positively associated with TB practices. A possible interpretation may be that people are more likely to adopt active TB prevention behaviours due to decreased immunity and an increased risk of TB with age [23].

The strength of this study is that it is the first study to investigate TB KAP among elderly people in China. The findings may provide targeted guidance for improving health policies. However, this study had some limitations. First, this study targeted elderly people in Bao’an District of Shenzhen; thus, the results are representative of elderly people only in the Shenzhen area instead of China. A survey of elderly individuals in multiple regions across the country is needed to further verify the results of this study. Second, the study population was selected from individuals who received health examinations at the selected health centres. This population already has access to care and may not be representative of those who do not undergo health examinations, which limited the generalizability of our data to other elder populations. Third, we excluded samples with more than 10% missing values from the data analysis. Those who had better memories were more likely to complete the questionnaire than others, which may lead to selection bias. Forth, the survey was cross-sectional, which limited interpretation of the temporality and causality of these findings. Final, other potential predictors, such as the accessibility of health services, social support, and a history of chronic disease, were not included in this study. More comprehensive and rigorous studies based on scientific sampling are needed.

## Conclusions

Elderly people in Shenzhen City had low levels of TB knowledge awareness and poor attitudes and practices regarding TB prevention and treatment. Furthermore, this study showed that elderly people with lower education levels and higher family annual incomes per capita had lower overall TB knowledge awareness rates and poor TB-related attitudes and practices. Therefore, the implementation of systemic health education and health promotion activities for elderly people targeting the transmission and curability of TB is recommended.

## Data Availability

Data may be made available by contacting the corresponding author.

## Abbreviations

TB: tuberculosis
WHO: World Health Organization
KAP: knowledge, attitudes, and practices
BMI: body mass index
