# Correction to: Analysis of the current status and associated factors of tuberculosis knowledge, attitudes, and practices among elderly people in Shenzhen: a cross-sectional study

**DOI:** 10.1186/s12889-021-11524-y

**Published:** 2021-08-05

**Authors:** Yunxia Wang, Yong Gan, Juanjuan Zhang, Jinzhou Mei, Jing Feng, Zuxun Lu, Xin Shen, Meigui Zhao, Yanfang Guo, Qing Yuan

**Affiliations:** 1 Shenzhen Bao’an Center for Chronic Disease Control, No. 332 Yu’an 2nd Road, Shenzhen, 518101 China; 2 Department of Social Medicine and Health Management, School of Public Health, Tongji Medical College, Huazhong University of Science and Technology, Wuhan, Hubei China

**Correction to: BMC Public Health 21, 1163 (2021)**

**http://orcid.org/10.1186/s12889-021-11240-7**

It was highlighted that the original article [1] contained some errors in the values in paragraph 4 of the Results section and in Table 2. This Correction article shows the correct values in this Results paragraph (in bold) and the correct Table 2.

**Table 2:** The distribution of participants’ responses regarding TB KAP (*n* = 1078)

Items	Responses	n	%
**Knowledge**			
Infectivity of TB	Yes	779	72.60
	No	83	7.74
	Do not know	211	19.66
Route of TB transmission	Touching items	85	7.94
	Sharing utensils	117	10.93
	Coughing or sneezing	332	31.03
	All of the above	319	29.81
	Do not know	217	20.28
TB symptoms	Headache or dizziness	25	2.35
	Coughing for longer than 2 weeks or coughing up blood	786	73.94
	Abdominal pain or diarrhoea	5	0.47
	Do not know	247	23.24
Prevention and control of TB	Covering mouth and nose when coughing or sneezing	184	17.18
	Wearing a mask	51	4.76
	Good nutrition	21	1.96
	Washing hands after touching items in public	48	4.48
	All of the above	767	71.62
Curability of TB	Yes	477	45.30
	No	277	26.31
	Do not know	299	28.40
**Attitudes**			
Do you think that TB is a terrible disease?	Yes	646	60.71
	No	225	21.15
	Do not sure	193	18.14
Would you like to learn about TB?	Yes	924	86.03
	No	15	1.40
	Do not sure	135	12.57
Would you like to participate in TB education activities?	Yes	921	86.89
	No	26	2.45
	Do not sure	113	10.66
Are you willing to complete treatment if you have TB?	Yes	261	24.90
	No	410	39.12
	Do not sure	377	35.97
Would you be willing to be screened for TB if you had suggestive symptoms?	Yes	387	36.44
	No	494	46.52
	Do not sure	181	17.04
**Practices**			
Have you ever taken the initiative to learn about TB?	Yes	354	33.08
	No	716	66.92
Would you urge your friends with suspicious TB symptoms visit the doctor?	Yes	649	60.54
	No	335	31.25
	Do not sure	88	8.21
Would you visit a health facility if you had a cough for more than 2 weeks?	Yes	903	84.08
	No	131	12.20
	Do not sure	40	3.72
Would you visit a health facility if you suspected that you had TB?	Yes	916	85.69
	No	132	12.35
	Do not sure	21	1.96
Would you stop spitting in public?	Yes	313	33.02
	No	580	61.18
	Do not sure	55	5.80
Would you cover your mouth and nose when coughing or sneezing?	Yes	265	25.09
	No	791	74.91

*Abbreviations*: *TB* Tuberculosis, *KAP* Knowledge, attitudes, and practices

**Results**

However, less than one-third of survey respondents (31.03%) knew the route of TB transmission. In terms of attitudes, most elderly people (**86.03% and 86.89%)** wanted to learn about TB or participate in TB education activities, respectively. More than one-third of respondents (**36.44%**) were willing to be screened for TB if they had suggestive symptoms. Only **225 (21.15%)** and **261 (24.90%)** participants did not think that TB is a terrible disease or were willing to complete treatment if diagnosed with TB.
